# *CIC::NUTM1* sarcomas occurred in soft tissues of upper limbs : a rare case report and literature review

**DOI:** 10.1186/s13000-024-01499-w

**Published:** 2024-06-08

**Authors:** Lina Zhao, Huihua He, Jiacai Ren, Yabing Huang, Honglin Yan, Jingping Yuan

**Affiliations:** Department of Pathology, Renmin Hospital of Wuhan University, Wuhan, Hubei 430060 China

**Keywords:** CIC, NUTM1, *CIC:NUTM1* sarcomas, NUT midline carcinoma, CIC-gene rearranged sarcomas

## Abstract

**Background:**

CIC-rearranged sarcomas (CRS) represent a new entity of undifferentiated small round cell sarcoma belonging to the Ewing-like sarcomas family. CRS are the most common type. Fusion partners for the *CIC* gene include *DUX4*, *FOXO4*, and the recently recognized*NUTM1*. Rare cases of *CIC::NUTM1* sarcoma in pediatric patients have recently been reported in brain, kidney, bone, and soft tissues. However, such cases have not been identified in the soft tissues of the limbs.

**Case presentation:**

We reported a case of *CIC::NUTM1* sarcoma located in the right upper limb of an 18-year-old man. The tumor displayed morphologic features typical of *CIC::DUX4* sarcomas, with small- to medium-sized round cells, a lobular pattern, focal spindling, myxoid stroma, and patchy necrosis. The tumor diffusely expressed NUTM1, was positive for WT1cter at weak to moderate intensity, and was focally positive for CD99, while it was negative for keratins, EMA, P40, MyoD1, myogenin, NKX2.2, BCOR, and pan-TRK. Fluorescence in situ hybridization analyses revealed cleavage of the *CIC* and *NUTM1* genes.

**Conclusion:**

*CIC::NUTM1* sarcomas represent a novel molecular variant of CRS with a preference for the central nervous system and younger pediatric persons. Its morphology and phenotype may be mistaken for NUT carcinomas, and the behavior is more progressive than other forms of CRS. For this rare and newly discovered gene fusion variant, it is necessary to integrate molecular and immunohistochemical findings with morphologic features in the diagnosis of undifferentiated neoplasms.

## Background

*CIC*-rearranged sarcoma (CRS) is an undifferentiated round cell sarcoma that was that was first reported by Kawamura-Saito et al. in 2006 [1]. Because the cellular morphology and immunophenotype associated with this disease overlap with those of Ewing sarcoma, CRS is also called Ewing-like sarcoma. CRS is the most common type of Ewing-like sarcoma, and it exhibits a more aggressive clinical behavior than Ewing sarcomas [2].

The *CIC* gene is located on chromosome 19p13 [3] and encodes capicua transcriptional repressor, which is a high-mobility group (HMG) box transcription factor. Established fusion partners for the *CIC* gene mainly include *DUX4* [4] and *FOXO4* [5]. However, a recent transcriptomic study of tumors of the central nervous system has identified *NUTM1* as a novel fusion partner of *CIC*. Importantly, *NUTM1* rearrangements are known to underlie NUT carcinoma (NC), a highly aggressive type of undifferentiated carcinoma that most commonly occurs in the thorax, head, and neck region along the midline. Almost 75% of cases of NC harbor a *BRD4::NUTM1* fusion originating from t(15; 19)(q14; p13.1) [6].

Gene profiles of tumors harboring *CIC::NUTM1* fusions cluster tightly with those of *CIC::DUX4*-related sarcomas, regardless of their anatomic location in the central nervous system, kidney, bone, or soft tissues [7, 8]. Compared to *CIC::DUX4* sarcomas, those with the *CIC::NUTM1* fusion tend to lead to a more aggressive clinical course, suggesting that this fusion is likely to be associated with worse prognoses, but additional clinical studies are needed to further investigate this hypothesis.

The morphologies of these various tumor types tend to be similar, thus potentially leading to misdiagnoses. For example, cells from sarcomas containing the *CIC::NUTM1* fusion exhibit similar morphologies as those from *CIC::DUX4* sarcomas, and the cellular morphology of *CIC::NUTM1* sarcoma overlaps with that of NC. In both cases, tumors have been found to contain sheets of discohesive cells, which range from round-epithelioid to spindle shaped, and the tumors tend to have collagenous or abundant myxoid stroma. As NC may also occur in soft tissues and visceral organs [9] and to overexpress the NUT protein, CRS has the possibility of being misdiagnosed as NC; therefore, further confirmation by molecular pathologic techniques such as fluorescence in situ hybridization (FISH) and/or RNA sequencing is required to confirm the diagnosis. We report herein the first case of a *CIC::NUTM1* sarcoma occurring in the soft tissues of the limbs. This case study identifies a new molecular subtype and raley location of the CRS.

## Case presentation

An 18-year-old male patient was diagnosed with *CIC::NUTM1*-containing sarcoma in the soft tissues of the limbs by the Department of Pathology, Renmin Hospital of Wuhan University. The diagnosis was confirmed independently by two senior professional pathologists.

**Fig. 1:**
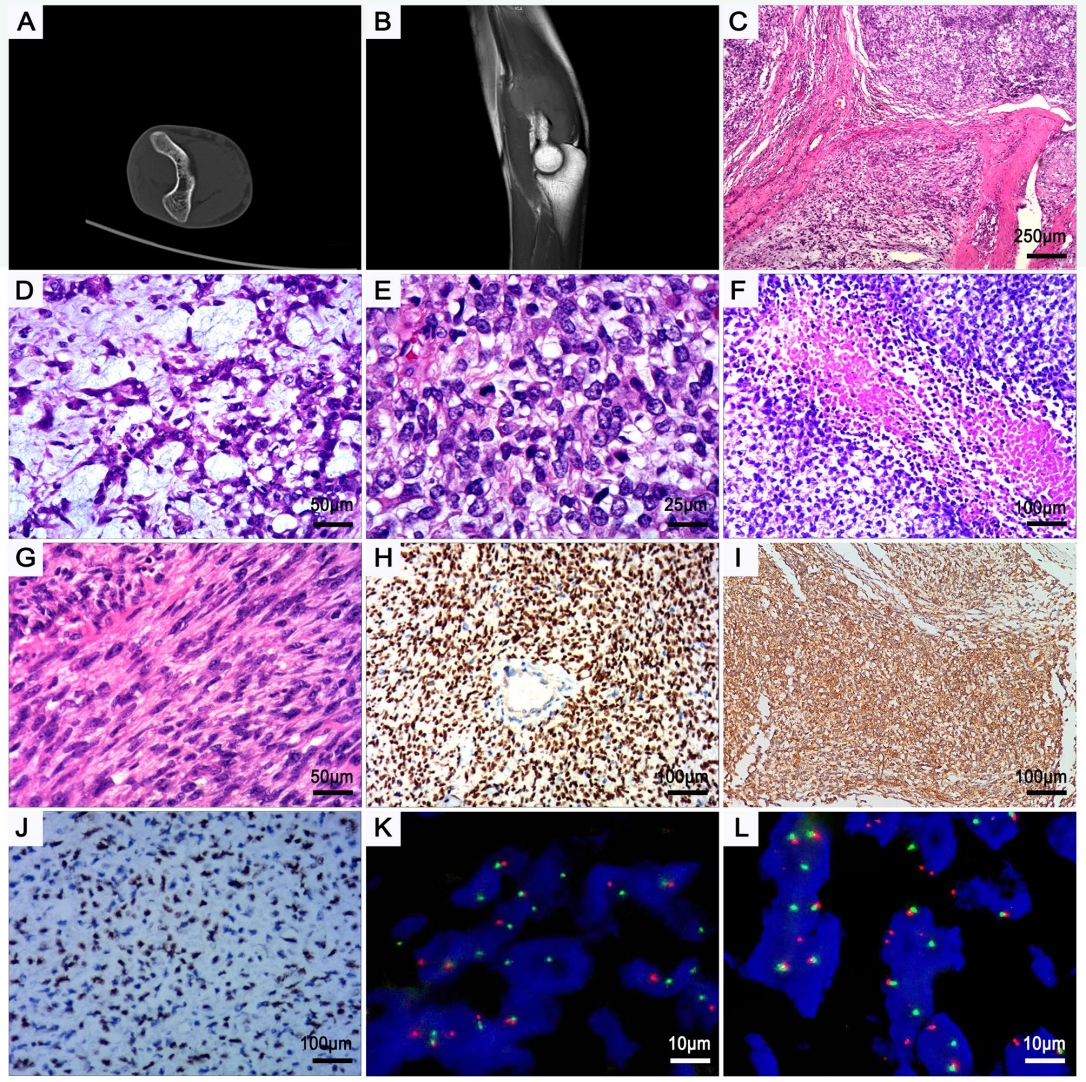
*CIC::NUTM1* sarcomas. (**A**), CT exam showed a soft tissue mass at the posterior part of the right distal humerus. (**B**), MR imaging showed soft tissue signal shadows behind the distal section of the right humerus. The boundary was clear from the surrounding fat space, and no bone erosion was observed. (**C**), The tumor consisted of solid sheets with a vague lobular form separated by fibrosis septa. (**D**), Myxoid stromal changes areas were focally present, classified as a “myoepithelial-like” growth pattern, with variable round and epithelioid appearance. (**E**), Under high power, the tumor cells exhibited scant cytoplasm, and the nuclei contained vesicular chromatin with prominent single nucleoli, and mitotic activity was brisk. (**F**), Large patchy necrosis was observed in the center of the nest. (**G)**, Tumor cells displayed spindles and were arranged in a storiform pattern. H to J, Immunohistochemistry showed diffuse nuclear expression of NUT (**H**) and nuclear and cytoplasmic expression of ETV4 (**I**), while staining was weakly positive for nuclear WT1cter (**J**). K and L, FISH using a CIC break-apart probe (**K**) and a NUTM1 break-apart probe (**L**)

The patient experienced pain in the arms for more than 6 months prior to hospital readmission due to persistent pain with progressive enlargement of a mass. Computerized tomography imaging (CT) showed a soft tissue mass at the posterior part of the right distal humerus with smooth boundaries and no obvious destruction of adjacent bones (Fig. 1A). Magnetic resonance imaging (MR) showed soft tissue shadow signals behind the distal section of the right humerus and high intensity but uneven signals inT1-weighted and T2-weighted short tau inversion recovery imaging (Fig. 1B).

The tumor size was approximately 6.0 cm × 1.9 cm × 4.8 cm, and it contained irregular nodules. The boundary was clear from the surrounding fat space, and no bone erosion was noted, leading to it classification as a neoplastic lesion. Thoracic CT demonstrated multiple masses in both lungs, which were suspected of being metastatic lesions.

### Morphology and immunohistochemical characteristics

Formalin-fixed paraffin-embedded tissue blocks were cut into 4-µm-thick sections and stained with hematoxylin and eosin using a standard protocol.

Upon gross examination, resection specimens presented as a solid mass that was grayish-white with hemorrhagic foci. The tumor margins were clear with focal fibrous pseudocapsules, and a local push-like infiltration into the surrounding skeletal muscle tissue was observed.

Histologically, the tumor presented an obvious nodular appearance, with nodules separated by thick fibrotic septa (Fig. 1C). The tumor consisted of uniformed small to medium sized round-to-ovoid cells with nuclei that were typically monotonous. The cells were arranged in a variety of patterns, mainly solid sheets or nests, with microcystic foci or loose reticular patterns in the chondroid or myxoid stromal background; these areas were similar in morphology to myoepithelial carcinoma in terms of the presence of loose or discohesive tumor cells (Fig. 1D).

The tumor cells exhibited scant cytoplasm that was transparent or mildly eosinophilic. The nuclei harbored hyperchromatic chromatin and vesicular chromatin with distinct single nucleoli. Cells exhibiting mitotic activity and apoptosis were relatively common, mitosis with approximately 19 observed per 10 HPFs (Fig. 1E). Large patchy necrosis was common (Fig. 1F).

The local area was composed of spindle cells arranged in herring bone or storiform patterns with rough nuclear chromatin was rough (Fig. 1G). The tumor cells expressed NUT (Fig. 1H) and ETV4 (Fig. 1I) proteins in a diffuse but strong and homogeneous pattern, and they were stained positive for WT-1 with weak to moderate intensity (Fig. 1J). The cell membranes exhibited multifocal strongly positive staining for CD99. Staining for P40, AE1/AE3, EMA, NKX2.2, myogenin, and MyoD1 were all negative. The Ki67 proliferation index was approximately 40%.

### FISH

Upon FISH examination of the tumors, *CIC* (Fig. 1K) and *NUTM1* (Fig. 1L) gene break-apart signals.

## Discussion

CRS is the most common subgroup of the Ewing-like sarcoma family. This subtype presents as an undifferentiated round cell neoplasm, characterized by the occurrence *CIC* gene rearrangements [4]. This gene encodes an HMG box transcription factor. *DUX4*, the most common *CIC* fusion counterpart, is a double homeodomain gene located on chromosome 4q35 or 10q 26.3 [2, 10]. The function of *DUX4* in normal cells remains unknown, but its expression is restrained in normal differentiated cells. The other, more rare, fusion gene partners ( including *FOXO4*, *LEUTX*, *NUTM1*, and *NUTM2A*) appear in approximately 5% of cases [5, 11, 12].

CRS mainly occurs in the soft tissues of young adults, especially in the soft tissues of the limbs and trunk. It also occurs rarely in older patients, where it is associated with a more aggressive clinical course than Ewing sarcomas [2]. CRS also shows a poor response to chemotherapy options that are used for Ewing sarcoma, and the 5-year overall survival rate is only 43%, which is significantly lower than that of Ewing sarcoma (77%) [2]. This biological evidence demonstrates that CRS represents a unique variant distinctive from Ewing sarcoma and it was acknowledged as an independent entity in the 2020 World Health Organization Classification of Bone and Soft Tissue Tumors (5th edition) [13].

The morphologic features of CRS include a lobular architecture divided by core fibrous septa, focal spindle cells, variable myxoid stroma, and extensive patchy necrotic areas. Compared with Ewing sarcoma, CRS is less monotonous and more pleomorphic. In particular, areas with vesicular nuclei and distinctive nucleoli are present in CRS. In rare cases, the neoplastic cells assume an epithelioid morphology, occasionally with rhabdoid cells or obvious cytoplasmic changes. The mitotic count is generally high. Most examples (85%) of CRS are found to express CD99 in various intensities [2], but in only 23% of cases is diffuse expression of CD99 observed. ETV4 [14] and WT1 (both WT1nter and WT1cter) are consistently positive [15].

NUT midline carcinoma is a clinically aggressive solid malignancy that mainly affects the axial structures of younger patients, and it typically results in death within a few months after diagnosis. The mediastinum and the upper aerodigestive tract are the most commonly affected anatomic locations [16]. With a propensity for early metastases to lymph nodes and pulmonary, the average overall survival of NC is only 6.7 months, and the prognosis is extremely poor [17]. *NUTM1* gene rearrangement is the typical pathogenic cause of NC, with the most common fusion partner of *NUTM1* being *BRD4* [16]. As a result, overexpression of the NUT protein is seen in the vast majority of NC cases, and it has been determined to be highly specific for NC [18]. Despite it is named “carcinoma”, the tumor may occur at any anatomic location, including soft tissue [19].

NC has recently been found to exhibit a wider morphologic spectrum than that previously associated with the disease. NC cells include small cells, epithelioid cells, and round cells of the myxoid and loose reticular type [9]. Nuclei are monotonous, showing a round-ovoid form. Mitotic activity is brisk, and necrosis is frequently present. Dickson et al. [9]. reported six cases of primary *NUTM1*-rearranged undifferentiated tumors that occurred in the soft tissue of the extremities, kidney, stomach, and brain, most of which contained sheets of discohesive cells ranging from round-rhabdoid-epithelioid to spindle shaped. The stroma in NC is collagenous or abundant myxoid, and these features have more morphologic overlaps with undifferentiated round sarcomas, including CRS and malignant myoepithelial tumors. No potent squamous keratinization has been found in any of these cases. Of the six cases reported by Dickson et al., four cases expressed cytokeratin, but only two cases were significantly positive, which differed from classical NUT middle carcinoma. The relationship between NUT-associated tumors of the soft tissue or viscera and NUT midline carcinoma remains unclear.

*CIC::NUTM1* sarcoma was initially identified in the central nervous system, but it may also occur in viscera, such as the kidney [20]. The sarcoma in the present case was located in the upper limbs, which was a rarely reported location. Nevertheless, bone involvement, especially spinal cord involvement [21], seems to be more frequent in *CIC::NUTM1* sarcoma when compared to *CIC::DUX4* sarcoma. Furthermore, patients with *CIC::NUTM1* sarcoma tend to be younger, mainly in the pediatric population, with a median age of 6 years [12] for NUTM1 as compared to an average age of 21.6 years for patients with *CIC::DUX4* (Table 1).

Tumors derived from this NUT fusion variant resemble NUTM1-rearranged undifferentiated tumors in soft tissue, and they tend to be associated with an aggressive clinical course. Antonescu et al. reported six cases of *CIC::NUTM1* sarcoma, one of which was in a locally advanced stage, with an average survival of 18.6 months, which was significantly shorter than the mean survival associated with *CIC::DUX4* sarcomas 139 months [2].

Our case progressed rapidly, with multiple bilateral lung metastases only 6 months after the first consultation. However, our case was found to be responsive to chemotherapy, as the primary tumor size shrank, and the metastatic pulmonary lesions were undetectable 6 months after the onset of chemotherapy. The primary tumor in the humerus also disappeared after 14 months of therapy, according to MR imaging. Therefore, while the *CIC::NUTM1* fusion type may be associated with worse prognoses, our experience with enhanced sensitivity to chemotherapy may indicate that future cases will have better prognoses than have previously reported cases. However, it is worth noting that the present study was limited to a single case, and the follow-up period was also limited. Accordingly, we believe that it will be important to accumulate additional cases and to extend the duration of follow-up in order to obtain a more comprehensive understanding of long-term outcomes and to draw more robust conclusions.

**Table 1:** Summary of clinicopathologic and molecular features of *CIC::NUTM1* sarcomas cases

Case No	Age, (y)/ sex	Location	Survival	Morphological findings		FISH CIC		FISH NUTM1		RNA- sequencing	NGS
1 [21]	61/M	C7-C78	DOD (8 mo)	Lobulated		Break		Break			CIC exon 16–NUTM1 exon 5
2 [21]	38/M	T7-T8	DOD (15 mo)	Solid and lobulated		Break		Break			CIC exon 17–NUTM1 exon 6
3 [2]	52/F	T11-T12	DOD (9 mo)	Solid and lobulated		Break		Break			CIC exon 17–NUTM1 exon 6
4 [12]	3/M	Temporal	DOD (18 mo)	Solid and trabeculae	Failure			Break		CIC(e18)- NUTM1(e5)	
5 [12]	5/M	Occipital	DOD (14 mo)	Solid	Break		Break		CIC(e17)- NUTM1(e5)		
6 [12]	7/M	T11-L2	DOD (37 mo)	Solid and lobulated	Beak		NA		CIC(e18)- NUTM1(e2)		
7 [12]	27/M	Left lung	DOD (7 mo)	Solid	Break		Break		CIC(e20)- NUTM1(e5)		
8 [12]	22/F	lateral ventricule	DOD (17 mo)	Solid and lobulated	Break		Break		CIC(e18)- NUTM1(e2)		
9 [12]	18/M	T9-T10	Alive (40 mo)	Solid and lobulated	Failure		Unbalanced break with gain of 3′ probe		CIC(e16)- NUTM1(e4)		
10 [24]	60/M	sphenoid	Alive (10 mo)	Solid and strand	Break		Break				CIC exon 17 -NUTM1 intron 4
11 [20]	13/F	renal	Alive (36mo)	Solid	Break		Break		CIC(e16)- NUTM1(e4)		
currentcase	18/M	upper Limbs	Alive (14 mo)	Solid and strand	Break		Break		Failure		Failure

*Abbreviations* F, female; M, male; C, cervical spine; DOD, died of disease; mo, month; FISH, fluorescence in situ hybridization; NGS, next-generation sequencing; T, thoracic spine

*CIC::NUTM1* sarcoma exhibits a similar morphology to *CIC::DUX4* sarcoma, including uniform small round cells arranged in a lobular pattern, with focal spindle cells and mucoid changes in the stroma. These characteristics are reminiscent of the myoepithelial-like features of *CIC::DUX4* sarcoma, and both exhibit distinct nucleoli [22]. Some cases of *CIC::NUTM1* are likely distinct from NC and are better classified as sarcomas, including those of the central nervous system or soft tissues [8]. Moreover, gene expression profiling performed in a large series of round cell sarcomas demonstrated that tumors with the *CIC::NUTM1* fusion clustered tightly with *CIC::DUX4* and *CIC::FOXO4*-positive sarcomas and separately from NCs [7], indicating a likely biologic relationship between these CIC-rearranged neoplasms; accordingly, the morphologies of these tumors more closely resemble those of classical CIC-fusion sarcomas. Our case showed classical morphologic features resembling those of *CIC::DUX4* sarcoma.

The immunohistochemical staining of NUTM1 normally exhibits diffuse and uniform nuclear staining, but it is negative in round cell sarcomas except for those involving *CIC::NUTM1*, and thus acts as a specific marker for this form of sarcoma. *CIC::DUX4* tumors consistently express ETV4 and WT1cter [15], because the *CIC::DUX4* fusion transcript induces upregulation of polyoma enhancer activator 3 (PEA3/ETV4/E1AF) at both the RNA and protein levels. Le Guellec [14] thus found that ETV4 could be used as a marker to identify CRS from other undifferentiated round cell sarcomas. Likewise, in a series of cases reported by Le Loarer, ETV4 was positive in all but one case involving *CIC::DUX4*, while it was negative in NC. Distinct from *CIC::DUX4* sarcomas, WT1cter is scattered positive in *CIC::NUTM1* sarcomas. Our case was consistent with *CIC::NUTM1* sarcomas: although the morphologic features presented similar to those of *CIC::DUX4* sarcomas, the immunophenotype showed weak intensity positive for WT1cter and diffuse nuclear staining of NUTM1 (Table 2).

NC may display a myoepithelioid appearance or the presence of chondroid differentiation in soft tissues, overlapping with the morphological features of *CIC-NUTM1* sarcomas; moreover, the immunophenotypes of both are NUTM1-positive, making distinguishing these tumors challenging.

**Table 2:** Differential diagnosis of *CIC::NUTM1* sarcomas

	Age (year)		Clinical site	Morphological characteristics	ETV4	NUT	WT-1	Mocecular	Survial(month)
*CIC::NUTM1* sarcoma	6 ~ 32	Bone often		Solid and lobulated	+	+	Weak+	CIC-NUTM1 gene fusion	18.6mo
NUT middle carcinoma	16 ~ 30	upper aerodigestive tract		Solid and trabeculae	-	+	-	NUT-BRD1 gene fusion	6.7mo
CIC gene rearranged sarcoma	6 ~ 81	Soft tissues		Solid and lobulated	+	-	-+	CIC-DUX4 gene fusion	139mo
Ewing sarcoma	<20	Bone		Diffuse	-	-	-	EWSR1-FLI1/ERG gene fusion	5-year survival rate over 90%

+, positive; -, negative; mo, month

**Fig. 2:**
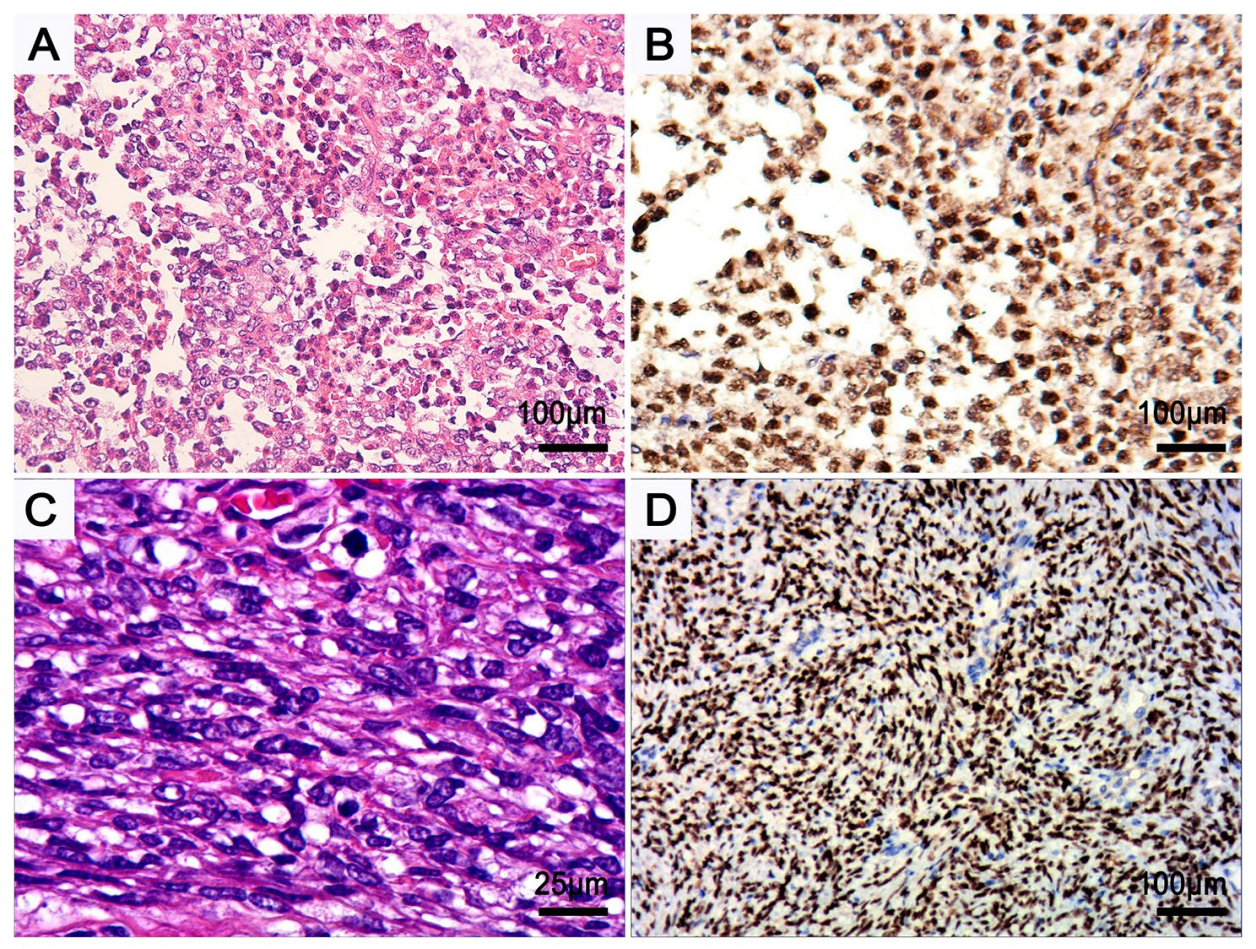
Differential diagnosis of NUT carcinoma versus *CIC::NUTM1* sarcoma. (**A**), NUT carcinoma in the mediastinum of a 23 year old female patient, with tumor cells arranged in sheets of epithelioid monomorphic cells with myxoid stroma. (**B**), NUT carcinoma typically exhibits a punctate-positive nuclear pattern. (**C**), *CIC::NUTM1* sarcoma with uniform small to medium cells of scant or clear cytoplasm. (**D**), NUT exhibits diffuse expression with a homogenous pattern in *CIC::NUTM1*, which is completely different from its expression pattern in NUT carcinoma

Positive staining for keratins and P40 does not necessarily permit a diagnosis of carcinoma over sarcoma. Moreover, cytokeratin is expressed consistently in several sarcomas, including epithelioid sarcoma and synovial sarcoma. Meanwhile, cytokeratin or P40 may be occasionally absent in NC [23]. Previous molecular studies have demonstrated that the *CIC::NUTM1* fusion (*n* = 3) clustered tightly with *CIC::DUX4* and *CIC-FOXO4*-fusion sarcomas and was split from NCs harboring the *BRD3/4::NUTM1* fusion, suggesting that all CIC-rearranged tumors belong to a unique family of tumors [7, 8]. Although NUTM1 is positive in both *CIC::NUTM1* and NC, the staining pattern is different. The diffuse homogeneous nuclear staining pattern of NUTM1 seems to be distinct from the dotted nuclear staining pattern in most NC (Fig. 2), although this distinction needs to be further examined in larger series. Therefore, the expression of ETV4 and homogeneous staining pattern of NUT leads to a preferential diagnosis of *CIC::NUTM1* sarcoma rather than a NUT carcinoma (Table 2).

## Conclusion

We report here an extremely rare case of a *CIC::NUTM1* sarcoma. This is the first case reported to occur in the soft tissues of the extremities, with genetic and clinicopathologic features distinct from those of CIC-rearranged sarcoma and NCs. Though the morphologic features of the tumors strongly resembled those of CIC-rearranged sarcomas, this entity showed a unique preference for axial bone and was associated with a more aggressive course, leading to a worse prognosis compared with classic CIC-rearranged sarcomas; therefore, we conclude that *CIC::NUTM1* sarcoma is a unique entity from classical CRS, and it should be considered to be different from other CRS. When a tumor shows the typical morphology of CRS but stains positive for ETV4 and shows a uniform nuclear staining pattern for NUTM1, clinicians should consider a diagnosis of *CIC::NUTM1* sarcoma. However, further confirmation by molecular pathologic techniques such as FISH or RNA sequencing should be employed to confirm the diagnosis.

## Data Availability

The original contributions presented in the study are included in the article. Further inquiries can be directed to the corresponding author.
